# The PGRS Domain from PE_PGRS33 of *Mycobacterium tuberculosis* is Target of Humoral Immune Response in Mice and Humans

**DOI:** 10.3389/fimmu.2014.00236

**Published:** 2014-05-27

**Authors:** Ingrid Cohen, Cristina Parada, Enrique Acosta-Gío, Clara Espitia

**Affiliations:** ^1^Departamento de Inmunología, Instituto de Investigaciones Biomédicas, Universidad Nacional Autónoma de México, Mexico City, Mexico; ^2^Facultad de Odontología, Universidad Nacional Autónoma de México, Mexico City, Mexico

**Keywords:** *Mycobacterium tuberculosis*, PE_PGRS33, PE domain, PGRS domain, latent tuberculosis infection

## Abstract

The PE_PGRS33 protein is a member of the PE family, which encompasses the PE and the PE_PGRS subfamilies. Among PE_PGRS’s, this protein is one of the most studied antigens and its immunomodulatory properties are influence by both PE and PGRS domains. However, the contribution of these domains to the host immune recognition of the PE_PGRS33 protein and their potential role in latent tuberculosis infection in humans is still unknown. In this study, the immunogenic properties of the complete PE_PGRS33 protein and each domain separately were evaluated in BALB/c mice and latent tuberculosis infected (LTBI) humans. In mice, PE_PGRS33 and its domains induced similar antibody production and secretion of IFN-γ. PE_PGRS33 and the PE domain stimulated higher CD4^+^ and CD8^+^ T-cell proliferation compared to the PGRS domain. This demonstrated that the principal difference in the immune recognition of the domains is the higher activation of T-cell subpopulations involved in the control of tuberculosis. In humans, the secretion of IFN-γ in response to PE_PGRS33 was detected in both LTBI and in non-infected vaccinated individuals. The same was observed for antibody response, which targets epitopes located in the PGRS domain but not in the PE domain. These observations suggest that T and B cell responses to PE_PGRS33 are induced by BCG vaccination and can be maintained for many years in non-infected individuals. This also indicates that the IFN-γ response detected might not be associated with latent tuberculosis infection. These results contribute to the elucidation of the role of the PE_PGRS33 protein and its PE and PGRS domains in the immune response against *Mycobacterium tuberculosis*.

## Introduction

*Mycobacterium tuberculosis*, the causative agent of human tuberculosis, is one of the most successful pathogens known. This bacterium is able to elude the host immune system and starts the disease after the infection or remains latent during long time (1). Many factors that could explain these characteristics were elucidated after the genome sequencing of the *M. tuberculosis* H37Rv (2). The genome sequence of these bacteria also revealed the presence of the PE family, which encompasses the PE and PE_PGRS subfamilies with about 100 genes scattered throughout the genome. Around 61 of these genes encodes for members of the PE_PGRS subfamily. These proteins are characterized by a highly conserved PE domain of approximately 110 amino acid residues that contains the motif Pro–Glu (PE) near the N-terminus. This domain is followed by the PGRS (polymorphic GC-rich-sequence) domain, which varies in size from 100–1400 amino acid residues and is rich in repetitive Gly–Gly–X motifs (2).

Some PE_PGRS proteins are exposed at the bacterial surface, where they can interact with the host immune system (3–5). Antibodies against PE_PGRS51, PE_PGRS62, PE_PGRS33, and the PGRS domain of Wag22 (Rv1759c^PE_PGRS^) are present in sera from patients with tuberculosis or during experimental tuberculosis in mice (6–10). Several PE_PGRS elicit T-cell responses in humans and are recognized by major histocompatibility complex-I (MHC-I)-restricted CD8^+^ T cells in mice, suggesting that many members of the PE_PGRS subfamily are highly immunogenic (11, 12).

PE_PGRS proteins are also involved in latency. Mutations in *pe_pgrs* genes of other mycobacterial species have shown decreased persistence in granulomas (13). The PE_PGRS33 protein is a member of the PE_PGRS subfamily that stimulates tumor necrosis factor-α (TNF-α) production, one of the cytokines involved in the induction and maintenance of latent tuberculosis infection in animal models mimicking human latency (14–16). The Rv1759c^PE_PGRS^ antigen induces immune response maintaining the latent infection in a murine model of chronic tuberculosis (17). *M. tuberculosis* clinical strains harboring big genetic variations in the *rv1818c* that codifies the PE-PGRS33 have been associated with clustering of tuberculosis cases and absence of cavitations in the lungs. This suggests that this protein plays a role in the establishment or maintenance of latent infection (18). Until now, the immune response against the PE_PGRS proteins has not been described in *M. tuberculosis* latent-infected individuals.

Additionally, the PE_PGRS33 protein plays an important and may be non-redundant role in the pathogenesis of *M. tuberculosis* (19). The sequence of the PE domain of this protein is highly conserved among *M. tuberculosis* clinical isolates (18, 20). This domain directs the cell wall localization of PE_PGRS33 (21). It has been reported that mutations in the PE domain affect the pro-inflammatory properties of the protein (22). On the other hand, the PGRS domain exhibits the major sequence variations in clinical *M. tuberculosis* strains (20). The PGRS fragment mediates the interaction with toll-like receptor 2 (TLR2) triggering host-cell death (14, 22). Deletions inside this domain can modulate the secretion of TNF-α induced by the PE_PGRS33 (14). The immunogenic properties of the PE domain have been evaluated in a murine model (9). The contribution of the PGRS domain to the immune response generated by PE_PGRS33 has been inferred from the study of the complete protein and the PE domain. However, the effect of the PGRS single domain has not been reported.

In this work, the immunogenic properties of the PE_PGRS33 protein and the PE and PGRS domains were studied in mice. This study was extended to humans where the secretion of IFN-γ and antibodies levels in latent tuberculosis-infected (LTBI) and non-infected individuals were evaluated.

## Materials and Methods

### Preparation of antigens

#### Cloning the PE and PGRS domains of PE_PGRS33

The full-length *rv1818c* gene, which codifies for the PE_PGRS33 was cloned into the pET15b vector (Novagen Inc., Madison, WI, USA) fused to a histidine (His) tag was kindly provided by Dr. M. J. Brennan [CBER, FDA, Bethesda, MD, USA (13)]. The *rv1818c* gene from pET15b was inserted into the plasmid pcDNA3 (Invitrogen, Carlsbad, CA, USA). An 1172 bp fragment (from nucleotide 339 to 1494) encoding the PGRS region of the *rv1818c* gene was amplified by PCR from pcDNA3 using the forward primer 5′-GGAATTCCATATGGGGCGCCCACTGATCGGT-3′ (which includes an NdeI site) and the reverse primer 5′-ATGGATCCCTACGGTAACCCGTTCATCCCGTTC-3′ containing a BamHI site and a stop codon. The NdeI–BamHI fragment was then cloned in pET15b. The PE coding region of the *rv1818c* gene (from nucleotide 1 to 339) was amplified using the forward primer 5′-CGGGATCCATGTCATTTGTGGTCACGATCC-3′ holding a BamHI site and the reverse primer 5′-CGGAATTCACAACAGCGCCAGGGCG-3′ including an EcoRI site and a stop codon. The EcoRI–BamHI fragment was then cloned into the multiple cloning site of the plasmid pGEX-4T-2 (Amersham Pharmacia, Piscataway, NJ, USA) to create a fusion product with the coding sequence for glutathione *S*-transferase (GST).

#### Expression and production of PE_PGRS33, PE, and PGRS domains

PE_PGRS33 and its PGRS domain were expressed in *Escherichia coli* C41 (DE3). Bacteria were cultivated at 37°C in Luria–Bertani broth with 100 μg/ml ampicillin. All the chemicals were obtained from SIGMA Aldrich, St. Louis, MO, USA unless otherwise stated. Protein expression was induced with 0.5 mM isopropyl-β-d-thiogalactopyranoside (IPTG) at OD = 0.6. Cells were harvested by centrifugation, resuspended in phosphate-buffered saline (PBS) with 1× complete ethylenediaminetetraacetic acid (EDTA)-free protease inhibitor (Roche Applied Science, Mannheim, Germany), and disrupted by sonication. After centrifugation, the inclusion bodies were washed sequentially with 2% Triton X-100, 1% Triton X-100, and PBS. The inclusion bodies were dissolved in buffer A [50 mM Na_2_HPO_4_ (pH 8.0), 8 M urea, 300 mM NaCl, 10 mM Imidazole] at 4°C for 14 h. The PE_PGRS33 protein was purified by metal affinity chromatography in an Akta-Prime (GE Healthcare Biosciences, Pittsburgh, PA, USA). The solution of proteins was bound to a His–Trap HP column (GE Healthcare Biosciences, Pittsburgh, PA, USA) previously equilibrated with buffer A. The protein elution was performed using 500 mM Imidazole in buffer. The purified protein was dialyzed and buffer was exchanged for 50 mM Tris–HCl (pH 8.0), 150 mM NaCl.

The PE domain fusion to GST was expressed in *E. coli* BL21 (DE3) grown in Luria–Bertani broth with 100 μg/ml carbenicillin at 37°C. Protein expression was induced with 0.25 mM IPTG at OD = 0.6. Cells were harvested by centrifugation and disrupted by sonication. Inclusion bodies were dissolved in 50 mM Tris–HCl (pH 8.0), 150 mM NaCl, 8 M urea, 1 mM dithiothreitol (DTT), 1 mM EDTA. The supernatant was dialyzed against 50 mM Tris–HCl (pH 8.0), 100 mM NaCl, and 1 mM DTT (union buffer) containing 4 and 2 M urea in sequential steps. The final dialysis step was performed against union buffer. A final concentration of 1% Triton X-100, 20 μg/ml PMSF, and 1 mM EDTA was added to the dialyzed sample. The suspension was bound to glutathione agarose at 0.5 ml resin per 2 ml sample by the batch method with gentle agitation at 4°C for 12 h. The resin was washed with 20 bed volumes of union buffer/1% Triton X-100 and then with 20 bed volumes of union buffer. The PE protein was eluted by incubation with elution buffer [50 mM Tris–HCl (pH 8.0), 100 mM NaCl, and 20 mM reduced glutathione] at 4°C for 90 min. The glutathione was removed by dialysis against 50 mM Tris–HCl (pH 8.0), 100 mM NaCl.

### Murine model

#### Preparation of PE_PGRS33 for the immunization of mice

A total amount of 100 μg of recombinant PE_PGRS33 was resolved in 12% polyacrylamide gels containing SDS according to the discontinuous buffer system of Laemmli (23). Proteins were transferred to nitrocellulose membranes (Amersham Pharmacia, Piscataway, NJ, USA). The protein band was identified by temporary staining with Ponceau S solution. The portion of the membrane with the identified protein was cut and converted into antigen-bearing particles using a previously described method (24). Briefly, the protein band was excised from the nitrocellulose sheet, cut in small pieces, and dissolved in dimethyl sulfoxide. The PE_PGRS33-bearing nitrocellulose was precipitated with 0.05 M carbonate/bicarbonate buffer (pH 9.6). After washing three times with PBS, the product was resuspended in 500 μl of sterile PBS. Nitrocellulose particles without protein were prepared under same conditions to be used as control. This methodology minimized the presence of undesirable proteins allowing the immunization to be performed only with the protein of interest.

#### Immunization of mice

The 6–7 weeks old female BALB/c mice were obtained from Harlan, Mexico. These mice were housed under standard pathogen-free conditions. All animal studies were carried out in strict accordance with the recommendations from the current Institutional Guidelines for the Care and Use of Laboratory Animals. The animal study was previously approved by the Committee for the Care and Use of Laboratory Animals of Instituto de Investigaciones Biomédicas. Two groups of mice (*n* = 4 per group) were used in each of three independent experiments. Mice were immunized by intraperitoneal injection of 20 μg of recombinant PE_PGRS33 on antigen-bearing nitrocellulose (100 μl of PBS containing 20 μg of protein per mouse). Control mice received 100 μl of PBS containing only nitrocellulose particles by the same route. Intraperitoneal booster injections with same amount of antigen-bearing nitrocellulose were administered on days 21 and 42 to immunized mice. Control mice were injected with nitrocellulose particles on days 21 and 42.

#### Carboxyfluorescein diacetate succinimidyl ester proliferation assay

Spleen cells were obtained from immunized and control mice 2 weeks after the last immunization. Cells were extracted by tissue disruption and suspended in RPMI 1640 medium. All chemicals were obtained from GIBCO, Gran Island, NY, USA, unless otherwise stated. The erythrocytes were lysed by mixing the cells with 0.15 M ammonium chloride (NH_4_Cl), 1.5 mM buffer HEPES (SIGMA Aldrich, St. Louis, MO, USA), 1 mM sodium bicarbonate (NaHCO_3_) at room temperature for 5 min. The reaction was stopped by adding 10 volumes of Dulbecco’s PBS (DPBS). Later 10^7^ splenocytes were incubated at room temperature in the dark with 0.5 μM carboxyfluorescein diacetate succinimidyl ester (CFSE, Invitrogen, Carlsbad, CA, USA) for 5 min. The reaction was stopped by adding nine volumes of RPMI 1640 plus 10% fetal bovine serum (FBS). CFSE-stained cells were centrifuged at 1500 rpm for 5 min, washed twice in DPBS with 10% FBS, and resuspended in RPMI supplemented with 10% FBS, 1.5 mM glutamine, 100 U/ml penicillin, 100 μg/ml streptomycin, 1% non-essential amino acids, 20 mM HEPES buffer, and 50 μM 2-mercaptoethanol. Viable cells were counted by trypan blue exclusion, and 1.5 million cells were stimulated with 25 μg of PE_PGRS33, PE, PGRS, or GST protein plus 10 μg/ml of polymyxin B (Calbiochem, Pacific Center, CA, USA) in 24-well plates at 37°C in 5% CO_2_ for 96 h. Polymyxin B was added to rule out the possibility of contamination with lipopolysaccharide (LPS). As positive control, 1.5 million CFSE-labeled spleen cells from control and immunized mice were stimulated with 2 μg/ml of concanavalin A (SIGMA Aldrich, St. Louis, MO, USA) at 37°C in 5% CO_2_ for 72 h. The cells were harvested, washed with DPBS with 2% FBS and 0.09% sodium azide (NaN_3_), and incubated with an anti-mouse FcγR antibody (CD16–CD32, CALTAG, Burlingame, CA, USA) diluted 1:500 to block the non-antigen-specific binding of conjugated antibodies. CFSE-labeled cells were then incubated with phycoerythrin-conjugated anti-CD4 antibody (BD Pharmingen, San Diego, CA, USA) diluted 1:200 and allophycocyanin-conjugated anti-CD8 antibody diluted 1:200 (CALTAG, Burlingame, CA, USA) on ice for 15 min. The cells were washed with DPBS with 2% FBS and 0.09% NaN_3_ and resuspended in 0.5 ml DPBS. Three-color flow cytometry was performed in a fluorescence-activated cell sorting (FACS) Calibur cytometer (BD, Mountain View, CA, USA). Lymphocytes and blasts were identified by forward scatter (FCS) and side scatter characteristics (SSC) in 10,000 events acquired. The percentage of proliferating cells was determined by gating on CD4^+^ or CD8^+^ cells and comparing the proliferating population (CFSE^dim^) with lineage positive cells that had not divided (CFSE^bright^). Cellquest™ software was used to acquire and analyze the data. Samples and controls were analyzed under same conditions.

#### Mouse cytokine assay

The supernatants from mice cultured spleen cells used in the CFSE proliferation assay were evaluated for IFN-γ production using a Murine IFN-γ ELISA development kit (PEPROTECH, Mexico City, Mexico) following the instructions of manufacturer. Briefly, ELISA plates containing 1 μg/ml capture antibody per well were incubated at 4°C overnight. After addition of standards and samples, plates were incubated at room temperature for 2 h. The detection was performed with 0.5 μg/ml detection antibody. The plates were incubated at room temperature for 2 h. Avidin peroxidase diluted 1:2000 was added and incubated at room temperature for 30 min. A color reaction was developed using ABTS liquid substrate and absorbance values were measured at 405 nm using an ELISA plate reader.

#### Antibody detection in mice

Two weeks after the last immunization blood was collected from the tail veins of the immunized and control mice. The titers of Immunoglobulin G (IgG), IgG1 and IgG2a in sera were determined using the ELISA assay. Briefly, 96-well plates (Maxisorp Nunc Immunoplates) were coated with 5 μg/ml of recombinant PE_PGRS33, PE, PGRS, or GST at 4°C overnight. The plates were blocked with bovine serum albumin (BSA. SIGMA, St. Louis, MO, USA) and incubated with different dilutions of the mouse sera (3 × 10^2^–1 × 10^5^). Goat anti-mouse IgG–horseradish peroxidase (HRP) conjugate, IgG1–HRP and IgG2a–HRP antibody (all from ZYMED, San Francisco, CA, USA) were used as secondary antibodies. A color reaction was developed with *o*-phenylenediamine tetrahydrochloride (SIGMA, St. Louis, MO, USA), and absorbance values were measured at 492 nm using an ELISA plate reader.

### Study in humans

#### Human donors

All the 88 volunteers who participated in this study were graduate students from the School of Dentistry, Universidad Nacional Autónoma de México. All participants had been vaccinated with *Mycobacterium bovis* Bacillus Calmette–Guérin (*M. bovis* BCG) when infants. The individuals were between 25 and 32 years old, clinically healthy, and had chest radiography negative for tuberculosis. The study in humans was performed in accordance with the Guidelines for Scientific Research with Humans of the Instituto de Investigaciones Biomédicas. The protocol was previously approved by the Ethics Committee of the same Institute. All participants signed consent forms detailing all relevant information about the nature of the study. All individuals participated voluntarily and their identities will remain undisclosed. No incentives were offered to participants.

#### IFN-γ release assay to detect LTBI and non-infected individuals

Blood samples were collected in heparinized tubes from each of the 88 participants. Aliquots of 1 ml of heparinized blood were incubated in the presence of the QuantiFERON^®^-TB Gold Kit (Cellestis Limited, Carnegie, VIC, Australia) antigens ESAT-6, CFP-10, and mitogen in 24-well tissue culture plates at 37°C in 5% CO_2_ for 16 h. Plasma was obtained by centrifugation and the samples were evaluated for IFN-γ production using the Human IFN-γ ELISA kit provided with the QuantiFERON^®^-TB Gold Kit following the instructions of manufacturer. Briefly, human IFN-γ standards and plasma samples were added to 96-well microplates coated with murine anti-human IFN-γ. The conjugate murine anti-human IFN-γ HRP was incorporated immediately after samples, standards, and conjugates were thoroughly mixed in a microplate shaker and incubated at room temperature for 2 h. After the addition of the enzyme substrate solution, the plate was incubated at room temperature for 30 min. The reaction was stopped with enzyme stopping solution. The optical density was read after 5 min using a microplate reader. The results were analyzed using the QuantiFERON^®^ Analysis software. The results of this analysis were used to divide the group of participants as LTBI and non-infected individuals.

#### IFN-γ release assay to detect responders to the PE_PGRS33 complete protein

An aliquot of 1 ml of heparinized blood obtained from each of the 88 participants was incubated with 25 μg/ml of PE_PGRS33 in 24-well tissue culture plates at 37°C in 5% CO_2_ for 16 h. Plasma was obtained by centrifugation and the samples were evaluated for IFN-γ production using the Human IFN-γ ELISA kit provided with the QuantiFERON^®^-TB Gold Kit following the instructions of manufacturer, as above described. The results of this assay identified responders to the complete PE_PGRS33 protein among the LTBI and non-infected individuals.

#### IFN-γ release assay to study the response to the PE_PGRS33 protein and its PE and PGRS domains in LTBI and non-infected individuals

A volume of 5 ml of blood was collected in heparinized tubes and diluted 1:10 with RPMI 1640 medium supplemented with 2 mM glutamine, 100 U/ml penicillin, and 100 μg/ml streptomycin (all from GIBCO BRL). An aliquot of 1 ml of diluted blood was incubated in a 24-well plate with 25 μg of PE_PGRS33, PE, PGRS, or GST protein plus 10 μg/ml polymyxin B at 37°C in 5% CO_2_ for 6 days. The concentration of IFN-γ in supernatants was quantified using the Human IFN-γ ELISA kit provided with the QuantiFERON^®^-TB Gold Kit according to the instructions of manufacturer, as above described.

#### Antigen-specific antibody detection in LTBI and non-infected humans

Antibodies against PE_PGRS33 and their domains were detected in 63 out of 88 sera of the individuals by ELISA assay. This assay was carried out as above described for antibody detection in mice with the following modifications: human sera were diluted 1:100, 1:300, and 1:500. All samples were incubated with anti-human IgG HRP conjugate. In addition, three samples from LTBI and four from non-infected individuals that produce IFN-γ in response to PE_PGRS33 were also tested with IgG1 HRP conjugates (Caltag, Burlingame, CA, USA). The color reaction was developed with *o*-phenylenediamine tetrahydrochloride. Absorbance values were measured at 492 nm using an ELISA plate reader.

### Statistical analysis

Data between groups were compared using the Mann–Whitney *U* test at the 0.05 significance level.

## Results

### Humoral and cellular immune response to PE_PGRS33, the PE, and PGRS domains in mice

#### Humoral immune response in mice

**Figure 1:**
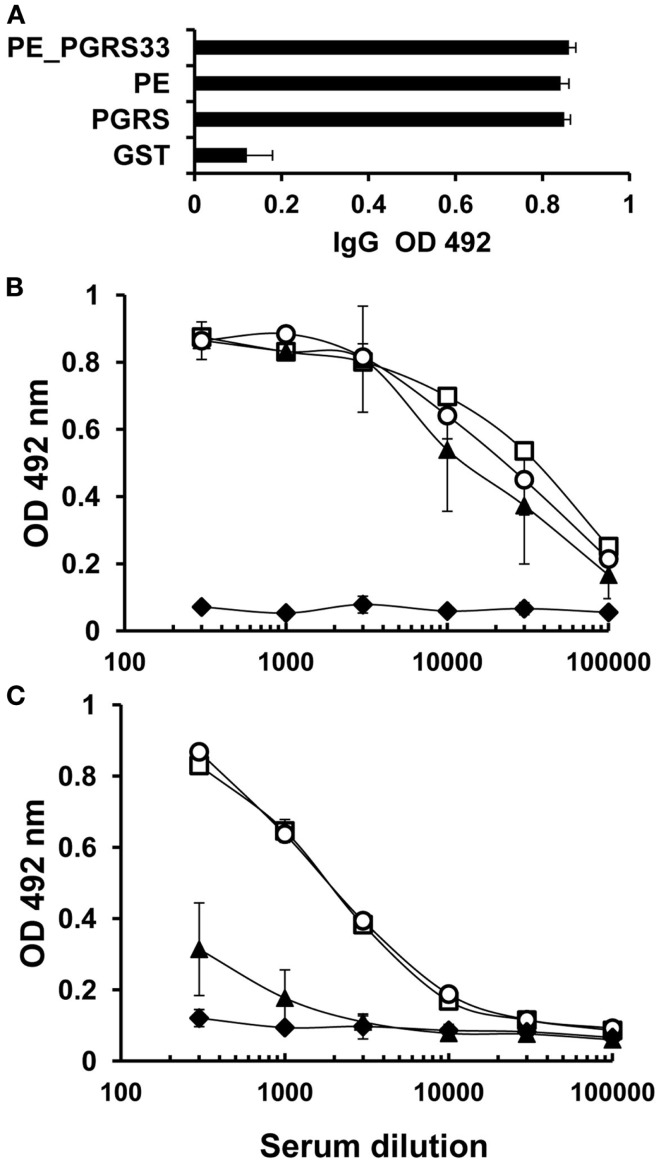
**Humoral immune response to PE_PGRS33, PGRS, and PE in immunized mice**. BALB/c mice were immunized with PE_PGRS33 and sera were collected 15 days after the last immunization. IgG **(A)**, IgG1 **(B)**, and IgG2a **(C)** responses to PE_PGRS33 (□), PE (▲), PGRS (○), and the control protein GST (♦), were evaluated by ELISA assay. For total IgG determination, each serum was diluted 1:100 and for IgG1 and IgG2a determination, mice sera were diluted as indicated in the figure. Bars **(A)** and datum points **(B,C)** are the average readings ± SD of data from four mice in the group, and the results showed are representative of three independent experiments.

Sera from immunized and control mice were tested using ELISA assay to determine the levels of antigen-specific IgG antibodies against PE_PGRS33 and its PGRS and PE domains. Similar titers of IgG were observed in both immunized and control mice (Figure 1A). Titers of IgG1 and IgG2a were quantified to identify the differences between these IgG subclasses. Similar titers of IgG1 against the complete protein and its domains were detected in all dilutions tested (Figure 1B). In contrast, the levels of IgG2a subclass against the PE domain were lower than the levels against the PGRS domain or the complete PE_PGRS33 protein (Figure 1C). This indicated that IgG1 antibodies in sera from the immunized mice targeted the complete PE_PGRS33 and its domains, whereas the IgG2a antibodies recognized epitopes located exclusively in the PGRS domain.

#### Antigen-specific T-cell proliferation in mice

**Figure 2:**
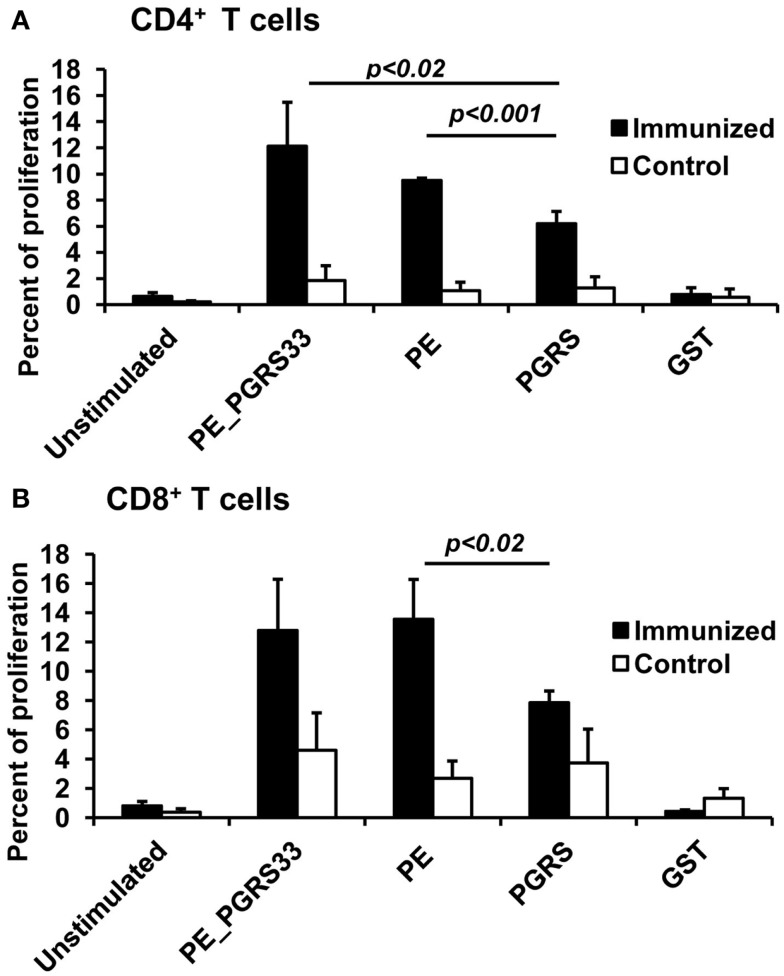
**Antigen-specific proliferation of CD4^+^ and CD8^+^ T cells in immunized mice**. Splenocytes from PE_PGRS33-immunized mice were stained with CFSE and incubated with 25 μg of PE_PGRS33, PGRS, PE, or GST protein plus 10 μg of Polymyxin B for 4 days. Spleen cells from mice injected with only nitrocellulose were also cultured with antigens (control). Cells without the antigens were incubated for the same length of time (unstimulated). Splenocytes were then labeled with anti-CD4-phycoerythrin **(A)** or anti-CD8-allophycocyanin **(B)** monoclonal antibodies and the percentage of proliferating cells were determined by CFSE dilution and flow cytometry. Each bar represents the mean ± SD of data from four mice per group, and the results are representative of those obtained from three independent experiments.

The proliferation of CD4^+^ and CD8^+^ T cells was measured in splenocytes of the mice immunized with the PE_PGRS33 protein to determine the contribution of PE_PGRS33 and its PE and PGRS domains to the activation of T cells (for flow cytometry histograms, see Figure S1 in Supplementary Material). The PE_PGRS33 complete protein similarly activated the proliferation of CD4^+^ and CD8^+^ T cells (Figure 2). Both the PE and PGRS domains stimulated the proliferation of CD4^+^ and CD8^+^ T cells. However, the response of the two cell subpopulations against the PE domain was significantly higher than that from the PGRS domain (Figure 2). The proliferative responses of lymphocytes from the control and immunized mice were statistically different (Figure 2). The GST co-expressed as a fusion product with the PE domain did not impact cell proliferation (Figure 2).

#### INF-γ secretion in mice

**Figure 3:**
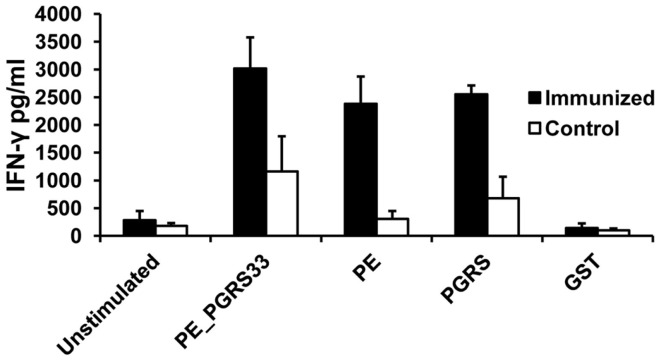
**Antigen-specific IFN-γ secretion in immunized mice**. Spleen cells from mice immunized with PE_PGRS33 or from control mice were incubated with 25 μg of PE_PGRS33, PGRS, PE, or GST recombinant purified proteins plus 10 μg/ml of polymyxin B for 4 days. The culture supernatants were evaluated for IFN-γ production using ELISA assay. Each bar represents the mean ± SD of data from four mice per group. Representative results of three independent experiments are shown.

The PE_PGRS33 protein, the PE, and PGRS domains induced significantly higher IFN-γ responses compared with unstimulated cells (Figure 3). The concentration of IFN-γ in the immunized mice was also higher than that from the controls (Figure 3). The levels of INF-γ secretion after stimulation with PE_PGRS33 and the domains were similar (Figure 3). This indicated that the PE_PGRS33, the PE, and PGRS domain are inducers of cellular immune response in mice.

### Immune response in humans

#### INF-γ responses to the complete PE_PGRS33 protein in LTBI and non-infected individuals

From the 88 individuals that participated in the study, 14 were identified as LTBI and 74 as non-infected (Table 1). To determine whether PE_PGRS33 is inducing an immunological response in humans, the secretion of IFN-γ by whole blood cells stimulated with the PE_PGRS33 protein was measured. The results obtained showed that the blood cells from 28.5% of LTBI secreted IFN-γ in response to the PE_PGRS33 compared to the 21.6% from the non-infected individuals (Table 1). This suggests that the IFN-γ response to the PE_PGRS33 protein is not associated with latent tuberculosis infection.

**Table 1:** **IFN-γ release assay to detect responders to the PE_PGRS33 complete protein in LTBI and non-infected individuals**.

Status of individuals	No. of individuals	% IFN-γ response to PE_PGRS33	
		Positive	Negative
LTBI	14	(4/14) 28.5	(10/14) 71.5
Non-infected	74	(16/74) 21.6	(58/74) 78.4

#### INF-γ response to PE_PGRS33 and its PE and PGRS domains in LTBI and non-infected individuals

From the 20 individuals (4 from LTBI and 16 from non-infected) that showed positive IFN-γ response to the PE_PGRS33 protein, only 7 were further tested for immune response to the PE and PGRS domains (Table 1). This group of participants that agreed to continue participating in the study included three LTBI and four non-infected individuals.

**Figure 4:**
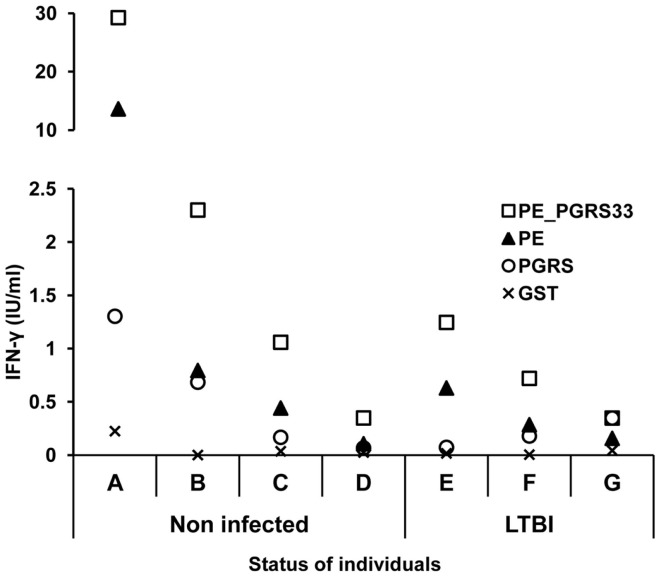
**INF-γ response to the PE_PGRS33 and its PE and PGRS domains in LTBI and non-infected individuals**. Diluted whole blood cells were stimulated with 25 μg of PE_PGRS33, PE, PGRS, or GST protein plus 10 μg/ml of polymyxin B for 6 days. IFN-γ in the supernatants was quantified using the Human IFN-γ ELISA kit provided with the QuantiFERON^®^-TB Gold Kit. Letters represent each individual tested and symbols correspond to the amount of IFN-γ produced by each of them.

The secretion of IFN-γ in response to the PE_PGRS33 protein and its PE and PGRS domains was quantified in whole blood cells from the seven participants with positive IFN-γ response to PE_PGRS33. The results indicated a higher secretion of IFN-γ in response to the PE_PGRS33 protein in non-infected individuals than in LTBI individuals (Figure 4). A tendency for IFN-γ secretion to be higher in response to the PE domain than the PGRS domain was observed (Figure 4).

#### Antigen-specific humoral immune response in LTBI and non-infected humans

**Figure 5:**
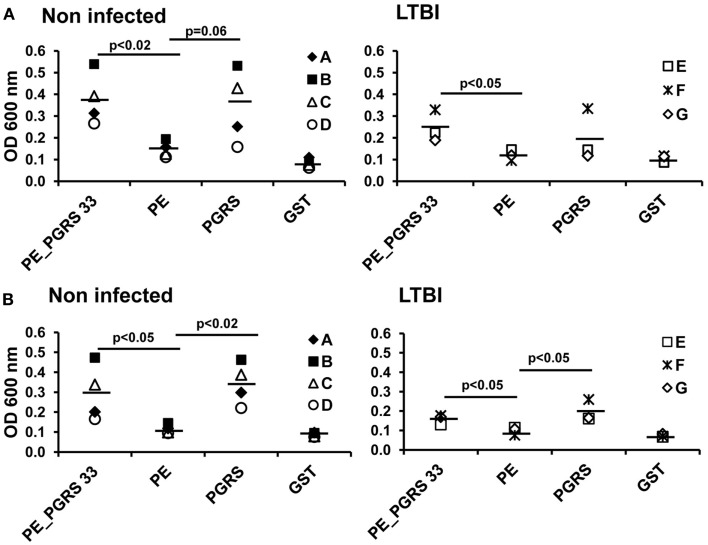
**Antigen-specific antibody response in LTBI and non-infected individuals**. Sera from LTBI and non-infected vaccinated individuals were diluted 1:300 and incubated with PE_PGRS33, PE, PGRS, and the control protein GST. Antigen-specific IgG **(A)** and IgG1 **(B)** were evaluated by ELISA. Letters in legend represent each individual tested and symbols correspond to OD readings. Mean values are showed as horizontal bars.

The serum from each of the seven individuals from LTBI and from non-infected individuals that produce IFN-γ in response to PE_PGRS33 was tested for antibody response using the ELISA assay. Human anti-IgG and IgG1 antibodies were used to detect specific antibodies against the PE_PGRS33 protein and its PE and PGRS domains. The IgG response against the PE domain was significantly lower compared to that from the PE_PGRS33 complete protein in the non-infected (*p* < 0.05) and in the LTBI individuals (*p* < 0.05) (Figure 5A). The IgG response against the PE was lower than that from the PGRS (*p* < 0.05) in non-infected individuals. On the other hand, the IgG response against the PE was as low as the response against the negative control (GST) in the LTBI individuals (Figure 5A). The IgG1 response against the PE domain was significantly lower than that against the PGRS domain and the PE_PGRS33 protein in non-infected and LTBI individuals (Figure 5B).

Furthermore, levels of IgG against PE_PGRS33 and their PGRS and PE domains were detected by ELISA in 63 sera from LTBI and non-infected individuals. Based in the above assay, only individuals with OD 600 nm over 0.3 were considered to have a significant amount of antibodies. It is worth of note, that 58.4% of non-infected individuals showed antibodies against the complete protein and a higher number of sera 66.6% recognized the PGRS domain. Results are shown in Table 2.

**Table 2:** **IgG levels against PE_PGRS33 and their PGRS and PE domains detected by ELISA in LTBI and non-infected individuals**.

Status of individuals	No. of individuals	% Individuals with IgG levels over 0.3 of OD 600 nm		
		PE_PGRS	PGRS	PE
LTBI	10	(2/10) 2	(4/10) 4	(0/10) 0
Non-infected	53	(31/53) 58.4	(35/53) 66.06	(0/53) 0

*Sera from LTBI and non-infected vaccinated individuals were diluted 1:300*.

Together these results indicated that the humoral immune responses against PE_PGRS33 targets epitopes mainly located in the PGRS domain.

## Discussion

The PE_PGRS33 protein has been involved in the pathogenesis of *M. tuberculosis* (18) and it is known that the PE domain is required for the protein translocation through the mycobacterial cell wall and the induction of primary necrosis (21, 25). The PGRS domain interacts with the TLR2-inducing apoptosis, targets the mitochondria triggering necrosis, and is responsible of the immunomodulatory properties of the entire protein (14, 22, 25). Even though the mentioned characteristics of the PE and PGRS domains have been elucidated, their contribution to the immunogenicity of the complete PE_PGRS33 protein has not been described.

In this study, the immunization of mice with the PE_PGRS33 protein stimulated CD4^+^ and CD8^+^ T-cell proliferation as well as IFN-γ secretion. This indicated that PE_PGRS33 is highly immunogenic. These results agreed with previous reports describing the immunogenic properties of the PE_PGRS33 (12). The CD4^+^ and CD8^+^ T cells are crucial in the protective host response against *M. tuberculosis*. These T-cell subsets migrate to the site of infection to produce the cytokines involved in the control of the disease (26, 27). The proliferation of CD8^+^ T cells caused by the immunization with the gene *rv1818c*, which codifies for PE_PGRS33 has been reported (12). The activation of CD4^+^ T cells in response to PE_PGRS33 presented in this work contribute to the knowledge of T cells subpopulations involved in the immunological response against this protein. These findings support the potential use of PE_PGRS33 as a vaccine candidate for tuberculosis (12).

The absence of secretion of IFN-γ in response to the full-length PE_PGRS33 protein has been reported in C57Bl/6 mice (9). In contrast, high concentrations of IFN-γ secreted using BALB/c mice were obtained. These results agreed with those published by Chaitra et al. (12) in same mice strain. The BALB/c mice were immunized with protein in the present study, while DNA and DNA prime-protein boosted were used in published works (12). Therefore, the immunization method does not explain the contrasting results. Such discrepancy is probably due to the dissimilar strains of mice used. This indicates that differences in the MHC might have an impact in the immune recognition of the PE_PGS33 protein.

The results of the cellular immune response in mice indicated that the PE and PGRS domains triggered the proliferation of CD4^+^ and CD8^+^ T cells. In agreement, Chaitra et al. (12) reported epitopes in the PE and PGRS domains presented to MHC-I and inducing effectors functions in CD8^+^ T cells. A MHC-II-restricted epitope found in the PE_PGRS53 is capable of stimulating CD4^+^ T-cell responses in human reactors to PPD (28). A comprehensive analysis of MHC-II epitopes has not been performed in PE_PGRS33. However, the activation of CD4^+^ T cells observed in this work suggested that both domains carry peptides inducers of MHC-II-dependent responses. According to the results obtained, both domains stimulated comparable IFN-γ secretion levels while the PE domain was the main inducer of proliferation of CD4^+^ and CD8^+^ T cells. This suggested that the PE domain could be stimulating a higher proliferation rate in these subpopulations to perform other functions besides the production of IFN-γ, as described previously (27).

When the PE_PGRS33 was used as immunogen in mice, the induced IgG levels to the full-length protein and to its domains was very similar. A possible explanation to this observation could be that when the domains are separated, they lose their original conformation and expose cryptic epitopes that are recognized by the antibodies generated in the immunized animal. On the other hand, in the complete protein these antigenic determinants remain hidden. This demonstrated that both domains are as highly antigenic as the entire protein in mice. The levels of IgG subclasses were further analyzed to detect differences in response to the domains studied. The IgG2a antibodies were directed to the complete protein and the PGRS domain whereas the IgG1 targeted all three antigens. The titration of these IgG subclasses revealed higher IgG1 levels than IgG2a. This showed that the IgG2a response is masked by the high IgG1 titer, which is the mayor contributor to the total IgG. These results indicated that PE and PGRS are antigenic in BALB/c mice with differences in the recognition at IgG subclass levels.

The role of some PE_PGRS proteins in mycobacterial persistence has been described (13, 17, 18). The identification of antigens interacting with the immune system during the latent infection will be essential in the development of immunological markers for this particular condition. One of the hypotheses of the present study was that PE_PGRS33 could be an important antigen in *M. tuberculosis* latency in humans. For this reason, a cellular immune response to PE_PGRS was expected in LTBI individuals. The results obtained in humans indicated that the IFN-γ response to the PE_PGRS33 protein might not be associated with latent tuberculosis infection. The high number of LTBI individuals with negative response to the PE_PGRS33 might be explained by the possible infection with strains not expressing *rv1818c* gene. Another reason could be the infection by strains containing large variations in the gene sequence. This genetic variation would result in significant changes in the PE_PGRS33 leading to the non-recognition by the immune system. Both mechanisms have been described to be a source of polymorphism for PE_PGRS members in clinical isolates of *M. tuberculosis* (20, 29–32).

The IgG1 subclass has been reported to be the predominant isotype in tuberculosis infection (33). For this reason, the humoral immune response to the PE_PGRS33 protein and the PE and PGRS domains was evaluated in LTBI and non-infected individuals by determination of total IgG and IgG1. The results showed that the antibody response was directed against the PE_PGRS33 protein targeting specifically the PGRS domain. The PGRS domain of PE_PGRS33 is rich in Gly–Gly–Ala–Gly–Gly repeats. These sequences could be the target of the antibody response observed in this study because proteins with repetitive amino acid sequences have been identified as immunodominant in rabbits and humans (7). In agreement, the PE_PGRS62 protein induced a strong antibody response against the full-length protein and a weak response to its PE domain in LTBI and non-infected humans (8). This supports the pattern of antibody recognition observed in this study for PE_PGRS33. The antibody response to the PE_PGRS33 in non-infected individuals can be attributed to *M. bovis* BCG vaccination. This indicates that sera reactivity to this protein in healthy individuals is independent of the infection with *M. tuberculosis*. In the PE_PGRS33 responders who participated in the second stage of the study, the cellular immune recognition of PE_PGRS33 showed a tendency to be higher in non-infected individuals compared with LTBI individuals. For the protein domains a clear tendency was not observed. In studies involving large populations a more evident trend might be obtained.

In conclusion, it was demonstrated that the PE and the PGRS domains have a role in the cellular and humoral immune response stimulated by the PE_PGRS33 protein in BALB/c mice. The PE_PGRS33 also induced the activation of T-cell subpopulations involved in the control of tuberculosis and secretion of IFN-γ. This confirmed the potential use of the PE_PGRS33 protein as candidate vaccine for tuberculosis and further increased the understanding of the immunogenicity of this protein. The IFN-γ response in humans to PE_PGRS33 protein might not be associated with latent tuberculosis infection. In this context, the PE_PGRS33 will not be suitable as immunological biomarker for this condition. The IFN-γ response and the sera reactivity to the PE_PGRS33 protein in healthy individuals is independent of the infection with *M. tuberculosis*. These observations suggest that T and B cell responses to PE_PGRS33 could be induced by BCG vaccination and can be maintained for many years in non-infected individuals. Additionally, the humoral immune response against PE_PGRS33 in humans targets epitopes located in the PGRS domain. All the findings reported here contribute to the elucidation of the role of the PE_PGRS33 protein in the immune response against *M. tuberculosis*.

## Author Contributions

Ingrid Cohen carried out the experimental procedures unless otherwise stated, performed statistical analysis, participated in the experiment design, and wrote the manuscript. Cristina Parada carried out ELISA assays in human studies and helped in mice experiments. Enrique Acosta-Gío participated in the design and coordination of the human sampling. Clara Espitia conceived and coordinated the study, participated in the experiment design, and wrote part of the manuscript. All authors read and approved the final manuscript.

## Conflict of Interest Statement

The authors declare that the research was conducted in the absence of any commercial or financial relationships that could be construed as a potential conflict of interest.

## Supplementary Material

The Supplementary Material for this article can be found online at http://www.frontiersin.org/Journal/10.3389/fimmu.2014.00236/abstract
