# Supplementary material for: The PGRS Domain from PE_PGRS33 of Mycobacterium tuberculosis is Target of Humoral Immune Response in Mice and Humans
Source: Front Immunol. 2014 May 27;5:236. doi: 10.3389/fimmu.2014.00236 (PMC4033847; doi:10.3389/fimmu.2014.00236)
Supplement: Supplementary file 1 [file Data_Sheet1.PDF]

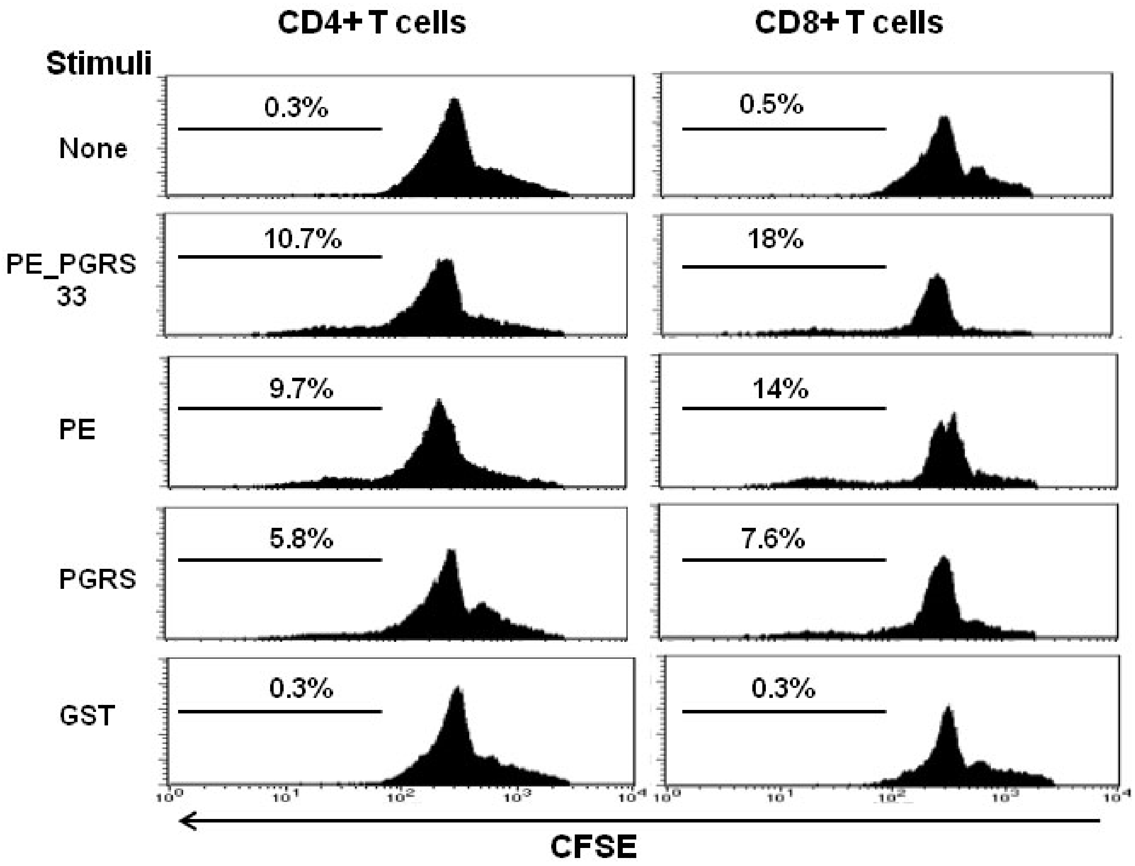

Supplementary Figure 1. Flow cytometry analyses of proliferating cells. Splenocytes from PE_PGRS33-immunized mice were stained with CFSE and incubated with PE_PGRS33, PGRS, PE or GST protein plus 10 µg of polymyxin B. Splenocytes were labeled with anti-CD4-phycoerythrin or anti-CD8-allophycocyanin monoclonal antibodies and cells were analyzed by flow cytometry. Lymphocytes and blasts were identified by forward scatter (FCS) and side scatter (SSC) characteristics. These cells were analyzed for CD4 and CD8 expression. $CD4^+$ or $CD8^+$ cells were gated and then a histogram was made to compare the proliferating population ($CFSE^{dim}$) with lineage positive cells that had not undergone cell division ($CFSE^{bright}$) after incubation with each stimuli.
